# Nanotechnology‐Enabled Blood Test for Oral Epithelial Disorders Management: A Proof‐of‐Concept Study

**DOI:** 10.1111/odi.15364

**Published:** 2025-05-06

**Authors:** Erica Quagliarini, Giulio Caracciolo, Francesca Giulimondi, Federica Rocchetti, Gianluca Tenore, Lucia Borghetti, Laura Ottini, Valentino Valentini, Antonella Polimeni, Umberto Romeo

**Affiliations:** ^1^ NanoDelivery Lab, Department of Molecular Medicine Sapienza University of Rome Rome Italy; ^2^ Department of Oral and Maxillofacial Sciences Sapienza University of Rome Rome Italy; ^3^ Department of Molecular Medicine Sapienza University of Rome Rome Italy

**Keywords:** biomarkers, blood test, nanoparticles, oral cancer, oral potentially malignant disorders, protein corona

## Abstract

**Objective:**

To address the limitations and complexities associated with tissue biopsy, emerging nanotechnology methods show promise in enhancing the detection and management of oral squamous cell carcinoma and oral potential malignant disorders. Among them, the characterization of the protein corona, the biomolecular layer that envelops materials when exposed to biological fluids, has recently emerged as a powerful tool for distinguishing oncological patients from healthy people.

**Materials and Methods:**

Building upon recent insights, here we formulated a new diagnostic method for oral squamous cell carcinoma and oral potentially malignant disorders based on the characterization of the personalized protein corona formed on graphene oxide nanosheets when exposed to the plasma of the patients.

**Results:**

The findings highlighted a discernible distinction between oncological subjects and healthy ones, resulting in an overall accuracy of 87%. Subsequently, we applied this method to monitor the progression of the diseases of the patients undergoing treatment, and we observed a trend indicating a convergence of protein profiles between oncological patients and healthy subjects throughout the treatment course.

**Conclusion:**

If further confirmed in larger prospective studies, this new approach could discriminate oral squamous cell carcinoma and oral potentially malignant disorders and can be considered in the management of these oral diseases.

## 1 Introduction

Oral squamous cell carcinoma (OSCC) represents a major global health problem, with an estimated > 300,000 cases and ~180,000 deaths per annum (Sung et al. 2021).

Major risk factors include tobacco smoking or chewing and drinking alcohol (Payaradka et al. 2022). Recently, the incidence of OSCC has been rising among young male non‐smokers and non‐drinkers due to the oncogenic role of human papillomavirus (HPV), especially type 16 (Isayeva et al. 2012). OSCC can arise de novo or from oral potentially malignant disorders (OPMDs) such as leukoplakia, proliferative verrucous leukoplakia (PVL), erythroplasia, oral lichen planus (OLP), and oral submucous fibrosis, with different potentials for malignant transformation (Nokovitch et al. 2023; Tenore et al. 2023). Currently, the diagnosis and treatment of OSCC represent a clinical challenge. To date, clinical examination of the oral cavity and biopsy of the suspected lesion followed by histological analysis are the gold standard for the diagnosis of OSCC (Mazumder et al. 2019). Several limitations should be acknowledged regarding tissue biopsy. Procuring biopsy material can be challenging at times due to the inaccessibility of certain tumors, post‐procedural physical discomfort, surgical complications, and a shortage of trained clinicians. Additionally, biopsy is temporally and spatially restricted, offering only a snapshot of a single region within a heterogeneous tumor (Palaia et al. 2022). Traditional approaches to managing OSCC include surgery, radiation therapy, chemotherapy, or a combination thereof, with the choice of therapy depending on the disease stage. Surgical resection currently serves as the standard recommendation, with a combined modality approach being favored for advanced disease (De Felice et al. 2014). However, this treatment approach often leads to significant complications, both physical and psychological. Indeed, OSCCs are frequently diagnosed at an advanced stage, contributing to a grim prognosis (with a mean 5‐year survival rate of less than 50%) (Warnakulasuriya and Kerr 2021). This poses a significant public health concern. Moreover, following primary treatment, recurrences and/or metastases occur in over half of the patients, with 80% of cases presenting within the first two years. Consequently, despite advancements in treatment, the annual mortality rate has remained stagnant, with 145,000 cases reported in recent decades (Cristaldi et al. 2019). Liquid biopsy is an appealing approach for the early diagnosis and monitoring of cancers, offering the possibility to introduce a noninvasive, fast, and cost‐effective method that could improve treatment and prognosis (Nonaka and Wong 2018; Di Santo et al. 2022; Temperini et al. 2022). Possible useful biomarkers are circulating tumor cells (CTCs), circulating tumor DNA (ctDNA) and circulating tumor RNA (ctRNA), proteins, and exosomes which can be found in several biological fluids such as blood, serum, plasma, saliva, pleural fluid, and urine (Peng et al. 2017). The evaluation of these cancer biomarkers in liquid biopsy has the advantage of providing a real‐time picture of primary and metastatic tumors at different time points, giving information about tumor burden and early evidence of drug resistance and tumor recurrence (Lousada‐Fernandez et al. 2018). However, the limited sensitivity of commercially available methods, including enzyme‐linked immunosorbent assay (ELISA), western blotting, flow cytometry, and mass spectrometry (SELDI‐MS or MALDI‐MS), coupled with the complexity and heterogeneity of the disease, hinders the widespread acceptance and dissemination of current tests (Liu et al. 2018; Soda et al. 2019; Iwano et al. 2021). In fact, efficient enrichment and high sensitivity are essential for effective biological detection since many biomarkers are present in low concentrations in body fluids. On the flip side, established techniques are labor‐intensive, costly, and time‐consuming, making them less adaptable for clinical applications, particularly for “point‐of‐care” tests (Boys et al. 2023). In fact, according to the World Health Organization (WHO), the experimental procedures for cancer screening and detection must satisfy the REASSURED (Affordable, Sensitive, Specific, User‐friendly, Rapid and robust, Equipment‐free, and Deliverable to end‐users) criteria (Land et al. 2019). In this context, promising prospects are emerging with the advent of nanotechnology in the realm of tumor diagnostics (Caputo, Quagliarini, et al. 2022). After decades of research dedicated to enhancing the efficacy and precision of cancer diagnostics, investigations into bio‐nano interactions have yielded valuable insights. Notably, it has been established that the protein corona (PC), the protein layer surrounding nanomaterials when immersed in a biological environment (Caracciolo et al. 2017), holds potential as a source of information pertaining to each individual's health spectrum (Caracciolo et al. 2014, 2019; Colapicchioni et al. 2016). This approach, which focuses on the personalized examination of the PC, carries significant implications for the field of tumor diagnostics. Indeed, the modulation of the PC is contingent upon various physicochemical properties of nanoparticles, such as size, shape, surface chemistry, zeta potential, etc., as well as the protein source (e.g., blood, gastric fluid, interstitial fluids, etc.) and environmental parameters (e.g., temperature, shear stress, etc.) (reviewed in Quagliarini et al. 2022). Thus, analytical processes aimed at monitoring PC composition present a novel avenue for the ex‐vivo capture of low‐abundance proteins in biological specimens, thereby augmenting the feasibility of detecting cancer biomarkers and offering insights into how the tumor affects the proteome and, ultimately, how it evolves over time. Among technologies under development, nanoparticle‐enabled blood (NEB) tests are emerging as a fast, cost‐effective, and user‐friendly tool for early cancer detection (Caputo et al. 2017; Papi et al. 2019; Caputo and Caracciolo 2020). NEB tests are based on the characterization of the PC formed on nanoparticles when exposed to healthy and disease‐affected patients, utilizing benchtop techniques such as dynamic light scattering (DLS), micro‐electrophoresis (ME), and one‐dimensional (1D) sodium dodecyl sulfate‐polyacrylamide gel electrophoresis (SDS‐PAGE) (Papi and Caracciolo 2018).

The aims of this study were (1) to investigate the possibility of creating and implementing a new technology based on NEB for the detection and management of OSCC and OPMD and (2) to explore the possibility of longitudinally monitoring OSCC and OPMD patients, seeking to correlate the outcomes of our diagnostic test with the response to treatment.

## 2 Materials and Methods

### 2.1 Study Cohort and Blood Samples

The study cohort was enrolled at the Department of Oral and Maxillofacial Sciences, MoMAX (Oral Medicine and Maxillofacial) ambulatory, from October 2022 to October 2023. The study was approved by the Local Ethical Committee (Sapienza University of Rome) of the Policlinico Umberto I of Rome with No. 0186/2022. All procedures performed in this study were in accordance with the ethical standards and/or national research committee and the 2002 Helsinki Declaration and its later amendments or comparable ethical standards. Written informed consent was obtained from all participants.

The inclusion criteria were patients with (1) a confirmed diagnosis of OSCC and OPMD based on both clinical and histological parameters; (2) age ≥ 18 years. The exclusion criteria were (1) patients who were not subjected to histopathological examination and/or (2) those with age < 18 years.

Patients were divided into three groups (G) according to pathology: G1 (*n* = 15), OSCC patients; G2 (*n* = 5), OPMD patients with mild or moderate dysplasia and G3 (*n* = 5), healthy controls. Patients underwent liquid biopsy from blood samples at diagnosis/enrollment (t0) and at 3 months (t1) and 6 months (t2) following disease management, except for controls that only underwent liquid biopsy at t0. At t0, G1 patients were 15, and G2 patients were 5. At t1 and t2, G1 and G2 patients were 5; 10 OSCC patients have not yet finished the follow‐up.

Patients who underwent liquid biopsy did not drink liquids, eat, or consume chewing gum in the 90 min prior to the biopsy.

Blood samples were collected in Blood RNA cfDNA/cf‐RNA 10 mL Preservative Tubes, containing the preservation reagent (Norgen, Biotek Corp.). The required volume of blood (8.4 mL) was stored at room temperature (16°C–24°C) and delivered within 48 h to the laboratory for plasma separation and molecular analysis.

### 2.2 Preparation of Graphene Oxide

Graphene Oxide (GO) was purchased from Graphenea (San Sebastian, Spain). GO was diluted in ddH2O at 1 mg/mL and subjected to sonication for 2 min at 125 W (Vibra cell sonicator VC505, Sonics and Materials, United Kingdom). GO water solution was characterized by dynamic light scattering with Zetasizer Nano ZS (Malvern, Herrenberg, Germany) while zeta‐potential measurements were performed using a Dip Cell Kit (ZEN1002). Data analysis was performed by averaging three measurements using Malvern Zetasizer software.

### 2.3 Preparation of GO‐PC Systems

To generate the personalized PC for each individual, appropriate volumes of GO solution (1 mg/mL) were incubated with low, medium, and high concentrations (i.e., 0.5% v/v, 5% v/v, and 20% v/v) of plasma derived from healthy and OSCC subjects and only at high concentration for OPMD subjects. Distilled water was added to each sample to reach a 100 μL final volume, then a 1‐h incubation at 37°C was performed to obtain GO‐protein corona systems.

### 2.4 Gel Electrophoresis Experiments

To eliminate free proteins from GO‐PC systems, samples were subjected to three centrifugations at 18,620 RCF for 15 min at 4°C; each time, the pellet was resuspended with ultrapure water. After the last washing step, the pellet was resuspended in appropriate volumes (i.e., 20 μL for low and medium plasma concentrations and 80 μL for high concentrations) of loading buffer (Laemmli loading buffer 1X), boiled at 100°C for 10 min, and centrifuged at 18,620 RCF for 15 min at 4°C. Finally, 10 μL of supernatant was loaded on a stain‐free gradient polyacrylamide gel (4%–20% TGX precast gels, Bio‐Rad, Hercules, CA, United States) (except for high plasma concentration, where 10 μL of supernatant was diluted 2.5 times before loading) and run at 100 V for about 150 min. Gel images were obtained with a ChemiDocTM imaging system (Bio‐Rad, Hercules, CA, United States) and were processed by ImageLab Software and custom Matlab (MathWorks, Natick, MA, United States) scripts to evaluate the one‐dimensional intensity distribution function of each sample and obtain the corresponding one‐dimensional molecular weight (MW) distribution.

### 2.5 Statistical Analysis

Data were analyzed using multivariate analysis by coupling the electrophoretic parameters (e.g., integral areas calculated from the one‐dimensional intensity protein profile) to differentiate healthy from oncological subjects. A one‐way ANOVA test was used for the significance with *p*‐values: *p < 0.05; **p < 0.001.

## 3 Results

The patient's clinical data are presented in Table 1. The gender distribution of OSCC patients was 8 males (53.3%) and 7 females (46.6%) with a mean age of 71.33. In most cases, 9 out of 15 (60%), the tumor was localized on the tongue, followed by the buccal mucosa and/or on the alveolar ridge or on the mouth floor. All cases of OSCC were grade 2 (G2); 4 patients were affected by OSCC stage I (26.6%), 2 patients are stage II (13.3%), 3 patients are stage III (20%), and 6 are stage IV (40%). According to the OSCC stage, patients affected by stage I/II were treated by surgical tumor resection while patients with stage III/IV were treated by surgical tumor resection and radiotherapy.

**TABLE 1:** Clinical characteristics of the patients are included.

Patients	Gender	Age	Type of lesions	Localization	Treatment
1	F	81	OSCC (Stage III)	Tongue	Surgical resection + Radiotherapy
2	M	68	OSCC (Stage I)	Tongue	Surgical resection
3	F	65	OSCC (Stage I)	Tongue	Surgical resection
4	M	76	OSCC (Stage IVa)	Alveolar ridge	Surgical resection + Radiotherapy
5	F	80	OSCC (Stage IVb)	Tongue	Surgical resection + Radiotherapy
6	M	86	OSCC (Stage II)	Alveolar ridge	Surgical resection
7	M	65	OSCC (Stage I)	Alveolar ridge	Surgical resection
8	F	81	OSCC (Stage IVb)	Buccal mucosa	Surgical resection + Radiotherapy
9	M	75	OSCC (Stage III)	Tongue	Surgical resection + Radiotherapy
10	M	56	OSCC (Stage IV)	Tongue	Surgical resection + Radiotherapy
11	F	62	OSCC (Stage IVc)	Tongue	Surgical resection + Radiotherapy
12	M	70	OSCC (Stage III)	Oral floor	Surgical resection + Radiotherapy
13	F	79	OSCC (Stage I)	Tongue	Surgical resection
14	M	53	OSCC (Stage IVb)	Tongue	Surgical resection + Radiotherapy
15	F	73	OSCC (Stage II)	Buccal mucosa	Surgical resection
16	M	77	PVL with mild dysplasia	Tongue, gingiva, hard palate, buccal mucosa	Laser vaporization and follow‐up
17	F	70	OLP with mild dysplasia	Tongue and buccal mucosa	Topical corticosteroids
18	M	65	PVL with moderate dysplasia	Hard palate and tongue	Laser vaporization and follow‐up
19	F	58	Oral lichenoid lesions with mild dysplasia	Buccal mucosa	Excisional biopsy
20	M	80	OLP with mild dysplasia	Buccal mucosa and tongue	Topical corticosteroids
21	M	61	Healthy control	/	/
22	F	72	Healthy control	/	/
23	F	48	Healthy control	/	/
24	M	52	Healthy control	/	/
25	F	80	Healthy control	/	/

The gender distribution of OPMD patients was 3 male (60%) and 2 female (40%), with a mean age of 70 years. Two patients (40%) were affected by OLP with mild dysplasia, two patients (40%) by PVL (1 with mild and 1 with moderate dysplasia) and 1 patient (20%) by lichenoid lesions with mild dysplasia. The most involved oral sites were the buccal mucosa and the tongue. Patients affected by OLP were treated with topical corticosteroids; patients affected by PVL were treated by laser vaporization and follow‐up, while the patient affected by lichenoid lesions was treated by excisional biopsy and dismissal of the cause.

In the healthy group, 3 were female (60%) and 2 were male (40%) with a mean age of 62.2 years.

### 3.1 Implementation of the NEB Test

**FIGURE 1:**
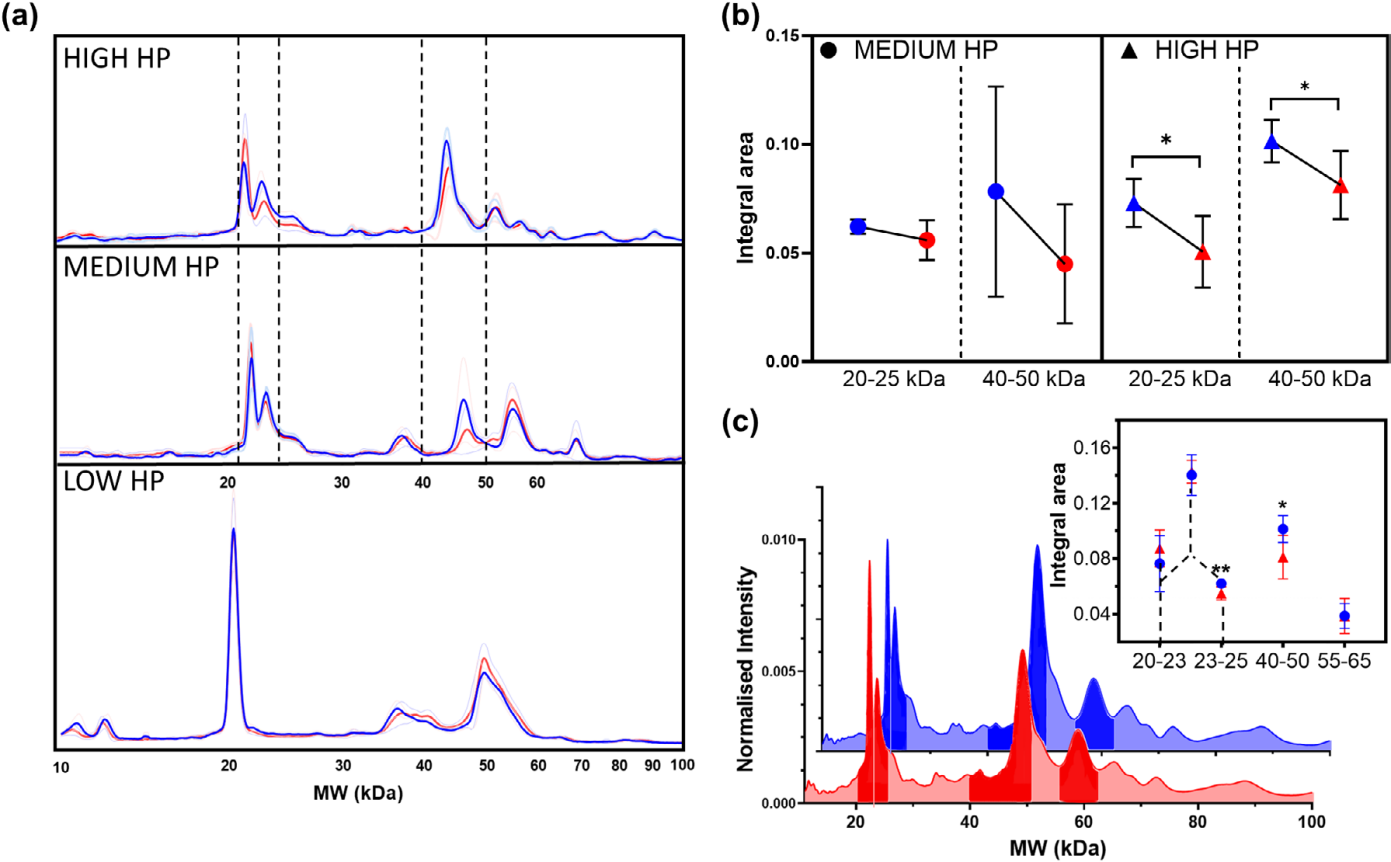
Protein corona characterization by gel electrophoresis. (a) Averaged intensity profiles calculated from the corresponding lanes of the SDS‐PAGE gel image arising from not oncological patients (NOP) (blue profile) and oral squamous cell carcinoma (OSCC) patients (red profile) at three different incubation concentrations, i.e., low, medium, and high (or 0.5%, 5%, and 20% v/v), among graphene oxide (GO) and donors' plasma. (b) Distributions of the integral areas calculated in the molecular weight (MW) interval between 20–25 kDa and 40–50 kDa derived from the profiles related to medium (circles) and high (triangles) plasma incubation conditions for NOP (blue) and OSCC patients (red). (c) Averaged intensity profiles calculated from the corresponding lanes of three distinct SDS‐PAGE gel images arising from NOP (blue profile) and OSCC patients (red profile) at high plasma conditions. In the inset, the distributions of the integral areas are calculated in the MW interval between 20–25 kDa, 40–50 kDa and 55–66 kDa and by splitting the first MW interval (20–25 kDa) in the two peak contributions, i.e., 20–23 kDa and 23–25 kDa. Asterisks correspond to a one‐way ANOVA test with *p*‐values: *p < 0.05; **p < 0.001.

**FIGURE 2:**
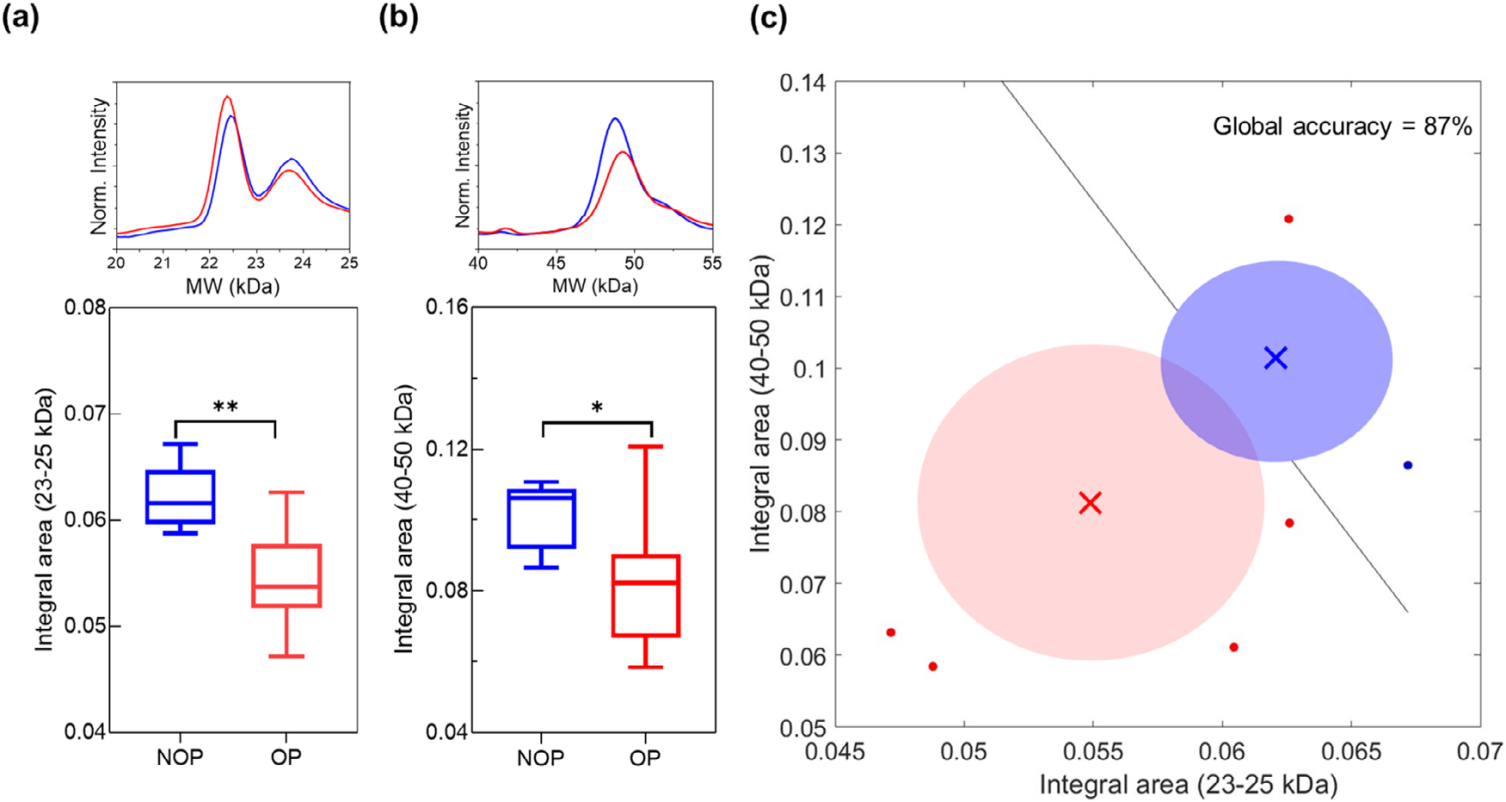
Comparison of protein profiles and discriminant analysis between oncological and not oncological patients. Averaged one‐dimensional protein profiles obtained by densitometric analysis performed on SDS‐PAGE gel images overlapped among not oncological patients (NOP) (blue curve) and oncological patients (OP) (red curve) and relative integral area distributions in the molecular weight between 23–25 kDa (panel a) and 40–50 kDa (panel b). (c) Linear discriminant analysis (black line) performed on the coupling between the two integral areas i.e., 23–25 kDa and 40–50 kDa, of NOP (blue dot) and OP (red dots) subjects, where the two class distributions are presented as confidence ellipses. By LDA computation, the resulting test's parameters read a global accuracy of 87%. Asterisks correspond to a one‐way ANOVA test with *p*‐values: *p < 0.05; **p < 0.001.

After achieving a homogeneous size by sonication of a GO water solution (1 mg/mL) for 2 min (Figure S1), the GO nanosheets were exposed to plasma at varying concentrations: low (0.5% v/v), medium (5% v/v), and high (20% v/v). The resulting PCs were subsequently analyzed using SDS‐PAGE. The gel image and two representative intensity profiles obtained from the gel analysis are presented in Figure S2. To facilitate a more comprehensive comparison among the three plasma conditions, we conducted a densitometric analysis of each gel lane. In Figure 1a, we have superimposed averaged intensity profiles derived from healthy donors (blue curves) and OSCC patients (red curves) in the molecular weight (MW) region between 10 and 100 kDa. We further compared the effects of the three plasma concentrations. Notably, the lowest plasma concentration (0.5% v/v) did not yield a clear discrimination between the two donor groups. Specifically, under this condition, the two intensity profiles completely overlapped across the entire MW region. However, as the plasma incubation amount increased, the protein profiles began to differentiate noticeably within the 20–25 kDa and 40–50 kDa intervals, as indicated by the dashed lines. Consequently, we conducted a more detailed analysis by calculating the integral areas relative to these two MW intervals under the two most discriminating plasma concentrations. In Figure 1b, the disparity between the integral areas of not oncological patients (NOP) (blue) and OSCC patients (red) is depicted, calculated from the MW region between 20–25 kDa and 40–50 kDa, at medium (represented by circles) and high (represented by triangles) plasma incubation conditions. As illustrated, the high plasma concentration revealed significant differences between healthy and oncological subjects in both the MW regions. These differences were supported by the *p*‐values around 4.5 × 10^−2^ from the one‐way ANOVA test. To enhance the robustness of the data, we conducted three distinct experiments at high plasma conditions. In panel c, the two profiles averaged over all three experiments for both the healthy subjects and the oncological subjects are presented. Within the profiles, we highlighted the MW areas with the most prominent peaks, which are likely to exhibit clearer distinctions between the two classes, i.e., healthy/diseased. These areas include the 20–25 kDa range, which presents two peaks (specifically between 20–23 kDa and 23–25 kDa), the 40–50 kDa range, and the 55–65 kDa range. Notably, from the analysis of the integral area distributions in the highlighted regions, it is evident that by discerning the contribution of the two peaks within the 20–25 kDa area, the difference in terms of integral areas allows for broader discrimination. Specifically, the significance shifted from a *p*‐value of 9 × 10^−2^ when the two contributions are not distinguished to a significance of 8.9 × 10^−3^, referring solely to the peak between 23 and 25 kDa. Finally, a significant difference was observed in the 40–50 kDa range, whereas no significant differences were found in the 55–65 kDa range. Then, we conducted a multivariate analysis by pairing the contributions associated with the most discriminant integral areas, namely the 23–25 kDa and the 40–50 kDa intervals, observed under the high plasma incubation condition. In Figure 2, we have included representative superimposed intensity profiles for NOP subjects (blue) and OSCC patients (red) in the two selected integral areas, along with the corresponding box plot distributions (23–25 kDa in panel a and 40–50 kDa in panel b). Additionally, we conducted a linear discriminant analysis (LDA) by integrating the contributions from these two areas. The linear discriminant analysis yielded a remarkable global accuracy of 87% (Figure 2c).

### 3.2 NEB Test for the follow‐up of the Patients

**FIGURE 3:**
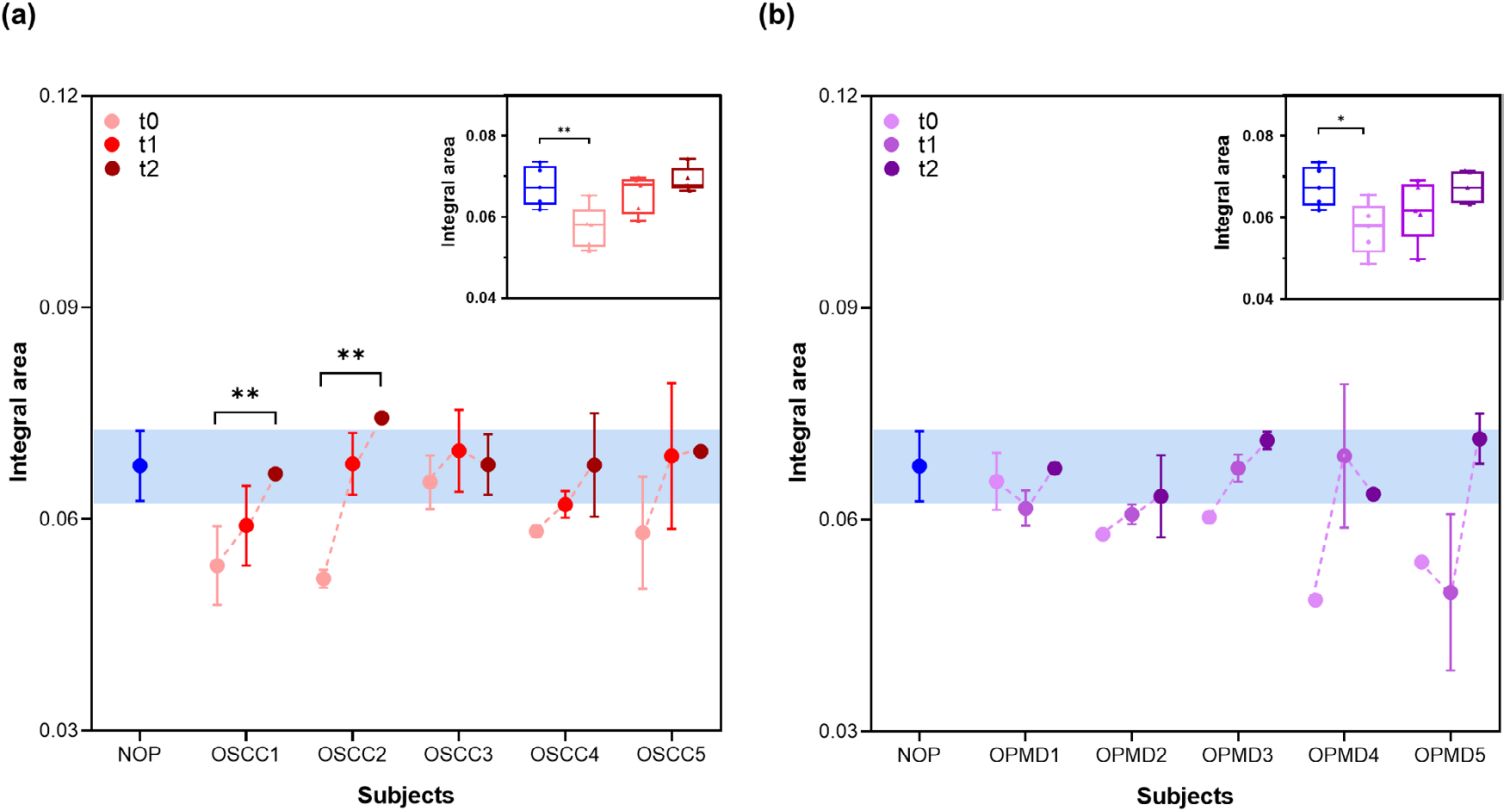
Follow‐up analysis developed from the electrophoretic profiles of the PCs of oncological patients. Representative trends over three time points of integral areas derived from the PC electrophoretic profiles in the MW range between 23 and 25 kDa, obtained from plasma samples of 5 oral squamous cell carcinoma (OSCC) subjects (i.e., OSCC1, OSCC2, OSCC3, OSCC4, and OSCC5) (panel a) and 5 subjects with oral potentially malignant disorders (OPMDs) (i.e., OPMD1, OPMD2, OPMD3, OPMD4, and OPMD5) (panel b). Samples were collected at t0 (initial collection), t1 (3 months post‐treatment for OSCCs and OPMDs), and t2 (6 months post‐treatment for OSCCs and OPMDs). Each data point represents the average integral area from three independent experiments on each subject, compared to the average of the corresponding integral area of the 5 not oncological patients (NOP). The insets display the integral area distributions of the 5 NOPs compared to the 5 OSCC patients (inset of panel a) and OPMD patients (inset of panel b), classified according to the three time points. Asterisks indicate results from one‐way ANOVA tests with *p*‐values: *p < 0.05; **p < 0.001.

As the second phase of our analysis, we employed the NEB test to monitor the progress of 5 subjects with OSCC and 5 subjects with OPMDs at the time of diagnosis/enrollment (t0) and at 3 months (t1) and 6 months (t2) following disease management (i.e., surgical treatment + radiotherapy or pharmacological treatment, respectively, for OSCCs and OPMDs). Firstly, we performed the NEB test based on SDS‐PAGE by incubating GO with the plasma of 5 NOPs and the 5 OSCC subjects at t0, t1, and t2 at the most discriminant plasma condition resulting from the preliminary analysis (i.e., 20% v/v, see ‘Implementation of NEB test’). In Figure 3a, we reported the trends of the integral areas calculated in the MW region between 23 and 25 kDa (emerged as the most distinguishing area) referred to each of the 5 OSCC patients (represented as OSCC1, OSCC2, OSCC3, OSCC4, and OSCC5) over the three‐time collections. The OSCC profiles were compared with the averaged integral areas of the 5 NOPs. As observed, the integral values of most of the oncological subjects progressively approach those of the healthy subjects, passing from t0 to t2. The boxplots featured in the inset of Figure 3a depict a thorough analysis of the integral area distributions for both the 5 healthy and 5 OSCC subjects, which these categorized based on the three distinct time points. It is worth noting that the distributions of OSCCs at t0 displayed significant differences from the NOP distributions. However, as expected, in the subsequent time points they converged with those of the healthy subjects.

In Figure 3b, we illustrated a comparison between the average integral area within the 23‐25 kDa range derived from 5 healthy subjects and the representative trends in integral areas across the three time points for the 5 patients with OPMDs (i.e., OPMD1, OPMD2, OPMD3, OPMD4, and OPMD5). Among the 5 OPMDs monitored, 2 (OPMD4 and OPMD5) showed a remarkable increase in the integral areas passing from t0 to t2, resembling values observed within the healthy subject threshold. Referring to the clinical data of these subjects, it appears that one patient was affected by lichenoid lesions with mild dysplasia and one patient by OLP with mild dysplasia. In the patients with lichenoid lesions, a complete excision of the lesions has been performed, which may justify returning the PC over time, similar to that of healthy subjects. In contrast, the patients affected by PVL did not show changes in the PC over time compared to healthy subjects, which indicates the presence of a risk of malignant transformation of these lesions.

## 4 Discussion

Despite the efforts performed to improve diagnosis, treatment, and prognosis, OSCC has been recently considered by GLOBOCAN as one of the leading causes of morbidity and mortality worldwide (Ferlay et al. 2015). The delay in OSCC screening and diagnosis may be the primary causative factor of this consideration (da Cunha Lima et al. 2021). In addition, the management of OSCC with the traditional strategies is not effective in some circumstances due to the dynamic behavior of OSCC with the modification of the molecular profile over time and due to the intratumoral and intertumoral heterogeneity (Irimie et al. 2018). Therefore, the recent literature is focused on trying to understand the molecular basis of OSCC as a trial to develop more accurate and custom‐made methodologies for early detection, prognosis, and establishment of successful therapies (Alix‐Panabières and Pantel 2021). Liquid biopsy with circulating biomarkers is one of these trials, which can be an opportunity for a better understanding of the profile and dynamic behavior of OSCC. This method has many advantages, such as easy accessibility, reliability, reproducibility, and the possibility for early detection and management of the disease. However, its introduction into daily clinical practice is not yet fully applicable due to its high cost, the low sensitivity, and the lack of a standard sample procedure (Zhang and Li 2023). In fact, biomarkers such as abnormally expressed proteins, peptide fragments, glycans, and autoantibodies from serum, urine, ascites, or tissue samples of cancer patients are present in a very low concentration (Steckl and Ray 2018). While proteomic technology advancement has facilitated their discovery, purification steps often result in loss of low molecular weight proteins that represent a potentially rich source of biomarkers (Huang et al. 2017). The latest developments in nanoparticle biotechnology have raised hope regarding the exploration of innovative and potentially impactful diagnostic methodologies. Thanks to their unique properties arising from their nanoscale dimensions, nanoparticles offer numerous tools for enabling earlier and more precise diagnosis, acting as nano‐accumulators of potentially relevant biomarkers. As a result, nano‐based systems are currently being exploited for the capture of various blood biomarkers, including proteins, DNA, and exosomes (Caputo, Coppola, et al. 2022; Di Santo et al. 2022, 2024). When a nanoparticle is introduced into a biological fluid, its surface promptly interacts with a variety of biomolecules, leading to a significant alteration of its synthetic identity. This bio‐nano interfacing gives rise to a dynamic biomolecular shell, predominantly composed of proteins referred to as PC (Pareek et al. 2018).

Over the years, it was demonstrated that the composition of the PC depends on three different categories of shaping factors (i.e., nanoparticle physical–chemical properties, protein source, and environmental factors) (Caracciolo et al. 2015). Among these, it was recently found that the concentration of the biological fluid is a determining factor involved in PC shaping, particularly for graphene‐based systems (Castagnola et al. 2018). For instance, Di Santo et al. explored the effect of the size of GO nanosheets and the concentration of plasma from healthy and pancreatic cancer‐affected patients on PC formation by 1D SDS‐PAGE experiments (Di Santo et al. 2020). Interestingly, they found that GO lateral size has a minor impact, if any, on PC composition, whereas protein concentration strongly affected the measured protein patterns. The PC has been successfully used for the detection of many validated biomarkers (Hajipour et al. 2014). Several studies were carried out and observe the feasibility of using nanoparticle–protein corona for early cancer detection. Azahar et al. employed graphene oxide–metal nanocomposites for the diagnosis of breast cancer by detecting epidermal growth factor receptors (ErbB2) (Ali et al. 2017). Colapicchioni et al. demonstrated the existence of a correlation between the plasma protein alterations in breast, gastric, and pancreatic cancers and the composition of the corresponding nanoparticle coronas (Colapicchioni et al. 2016).

Based on these recent findings, we formulated a diagnostic approach centered around the analysis of the personalized PC that formed on GO nanosheets when exposed to the plasma of patients affected by OSCC. To our knowledge, this is the first study conducted on the application of PC on oral cancer. We demonstrated that this approach benefits from the existence of several tunable parameters, particularly we found that the concentration of biological fluid exposed to GO is a critical factor shaping the PC profiles of OSCC subjects. Out of the three plasma concentrations examined, the highest concentration led to a notable distinction among the protein profiles of healthy individuals and those with OSCC, correctly classifying 18 out of 20 subjects. While a straightforward interpretation is beyond the scope of this investigation, we speculate that proteome alteration resulting in distinct protein patterns from plasma of OSCCs occurred within low MW proteins ranging from 20 to 30 kDa and 40–50 kDa. Undoubtedly, a qualitative approach such as mass spectrometry experiments would enable a more comprehensive investigation into the identity of potential protein biomarkers present in these regions. However, we see this result as the proof of concept that exposing nanomaterials to plasma samples under “ideal dilution conditions” is a good strategy to amplify personalization of the PC and, in turn, to exploit it to distinguish between healthy and OSCC patients. Although the limited sample size prevents definitive conclusions, we construed this result as a potential springboard for more in‐depth research studies that can foster personalized and cost‐effective healthcare provisions for patients with OSCC. Thus, the NEB test optimized on the OSCC cohort was used to track the advancement of five subjects diagnosed with OSCC after undergoing surgical treatment over two‐time points. As expected, the observed distributions of OSCC before clinical intervention exhibited notable distinctions from the distributions observed in healthy individuals. However, these distributions gradually aligned with those of the healthy subjects at subsequent time points. This finding underscores the dynamic nature of protein expression patterns in response to clinical interventions, providing valuable insights into the feasibility of using the NEB test for monitoring OSCC patients. Certainly, further research with larger cohorts is warranted to validate these observations and elucidate the underlying mechanisms driving these changes.

Ultimately, the integration of the NEB test into point‐of‐care tests is a promising approach to enhancing the precision and efficacy of tumor prediction in individuals at a heightened risk of tumor development. Our last effort was to apply our NEB test based on PC characterization to evaluate 5 subjects with potentially malignant lesions at three different time points (t0 before clinical treatment and t1 and t2 after treatment). Potentially malignant lesions assume a critical role as predictive indicators in the onset of malignancies, including OSCC (Warnakulasuriya 2020). These anomalies, characterized by atypical tissue changes predisposed to malignant transformation, serve as initial markers of potential cancer progression (Speight et al. 2018). The monitoring of OPMDs in clinical practice is pivotal for implementing timely and personalized interventions aimed at preventing the progression of OSCC. The high sensitivity of the NEB test may enable the identification of subtle biomolecular changes associated with the advancement of malignant lesions, facilitating prompt intervention and personalized treatment approaches.

Notably, among the monitored OPMD cases, two patients (OPMD4 and OPMD5) exhibited a significant distinction in PC profile after intervention, resembling values observed within the healthy threshold, as evidenced by the convergence of the integral areas derived from the PC profile in the MW range between 23 and 25 kDa with those of healthy individuals. Such alterations in integral areas suggest dynamic changes in the protein composition in the low MW proteins, which may hold diagnostic or prognostic significance in OPMD regression. In contrast, patients with PVL did not show changes in the PC. A systematic review with meta‐analysis by Palaia et al. reported that in a total of 699 PVL patients, 320 developed oral verrucous carcinoma (OVC) or OSCC (45,8%), emphasizing the risk of malignant transformation of this lesion over time and the need for frequent follow‐up (Palaia et al. 2021).

There are some limitations that should be acknowledged for the proper interpretation of our results, including the limited sample size and the lack of analysis of some OPMDs, such as leukoplakia and erythroplakia.

It is believed that the conventional tissue biopsy is still essential for definitive diagnosis and treatment planning, while the NEB test would be helpful in providing information for monitoring the progress of the disease, the treatment outcome, and the eventual risk of recurrence, with the advantage of avoiding repeated biopsies for these issues.

Overall, we envision that investigating the interplay between nanomaterials and plasma from OSCC and OPMD patients will present unparalleled prospects for advancing intricate in vitro diagnostic (IVD) technologies for this disease, applicable across all stages of patient care, spanning from initial diagnosis to disease progression tracking and personalized treatment evaluation.

Considering the potential for further, more in‐depth research studies, it is worth noting that the versatility of our method paves the way for future investigations on other biological fluids, such as saliva. Opting for saliva as the foundation for a detection technology may offer several advantages over plasma. Saliva collection is non‐invasive, ensuring a more comfortable experience for individuals. Its simplicity and ease of handling make it a cost‐effective and accessible option. Reduced contamination risk, improved patient compliance, and the dynamic biomarker composition of saliva enhance its potential for detecting and monitoring conditions. Additionally, real‐time monitoring capabilities make saliva a compelling choice for certain detection technologies. Thus, future investigations will be directed toward employing saliva as the primary biological fluid, reflecting a strategic focus on its non‐invasive nature and accessibility for advanced diagnostic investigations.

## Author Contributions

**Erica Quagliarini:** conceptualization, investigation, writing – original draft, writing – review and editing, formal analysis, data curation, validation, resources. **Giulio Caracciolo:** conceptualization, writing – review and editing, supervision, validation, resources. **Francesca Giulimondi:** investigation, writing – review and editing, data curation. **Federica Rocchetti:** conceptualization, investigation, writing – original draft, writing – review and editing, data curation, validation, resources. **Gianluca Tenore:** conceptualization, writing – review and editing, supervision, validation. **Lucia Borghetti:** investigation, data curation. **Laura Ottini:** writing – review and editing, investigation, methodology, data curation. **Valentino Valentini:** conceptualization, investigation, supervision. **Antonella Polimeni:** conceptualization, supervision, project administration, validation, resources. **Umberto Romeo:** resources, supervision, validation, conceptualization, investigation, methodology.

## Ethics Statement

All procedures performed in this study were in accordance with the ethical standards and/or national research committee and the 2002 Helsinki Declaration and its later amendments or comparable ethical standards.

## Consent

Informed consent was obtained from all the patients involved in the study.

## Conflicts of Interest

The authors declare no conflicts of interest.

## Supporting information


**Figure S1.** Physicochemical characterization performed by dynamic light scattering (DLS) in terms of Z average, Z potential, and polydispersity index (PdI) of graphene oxide (GO) nanosheets in water solution (1 mg/mL) after 2 min of sonication. Data are reported as average ± standard deviation of three independent measurements.

**Figure S2.** After exposing graphene oxide (GO) sheets to human plasma (HP), a protein corona (PC) is generated. Following isolation of the PC through centrifugation and subsequent analysis using 1D SDS‐PAGE, an image of the gel is produced. In the gel image, each lane corresponds to the protein profile derived from an individual human subject, either a notoncological patient (NOP) or a patient affected by oral squamous cell carcinoma (OSCC). Representative 1‐dimensional (1D) profiles were derived by performing densitometric analysis on the two gel lanes highlighted by dashed boxes in the gel image. These profiles are presented for NOP (blue profile) and OSCC (red profile) within the molecular weight (MW) range of 10 to 100 kDa.

## Data Availability

Data available on request from the authors.
