# Supplementary figures and images for: Nanotechnology‐Enabled Blood Test for Oral Epithelial Disorders Management: A Proof‐of‐Concept Study
Source: Oral Dis. 2025 May 6;31(11):3039–48. doi: 10.1111/odi.15364 (PMC12803622; doi:10.1111/odi.15364)

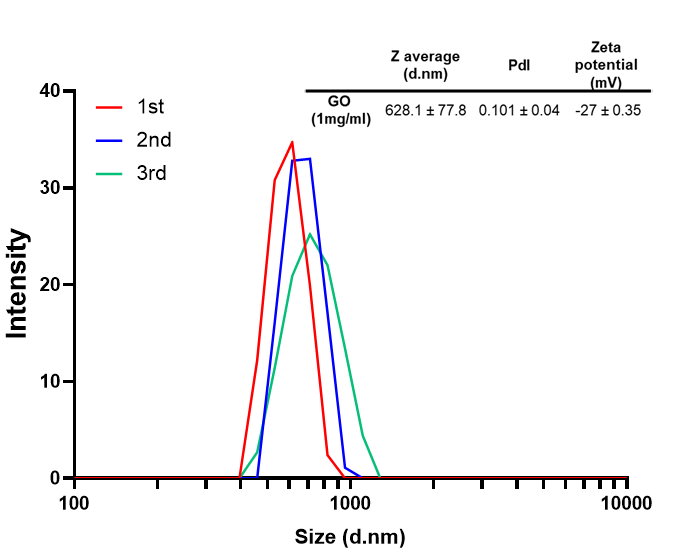

Supplement: Supplementary file 1 — Figure S1. Physicochemical characterization performed by dynamic light scattering (DLS) in terms of Z average, Z potential, and polydispersity index (PdI) of graphene oxide (GO) nanosheets in water solution (1 mg/mL) after 2 min of sonication. Data are reported as average ± standard deviation of three independent measurements. [file ODI-31-3039-s001.png]

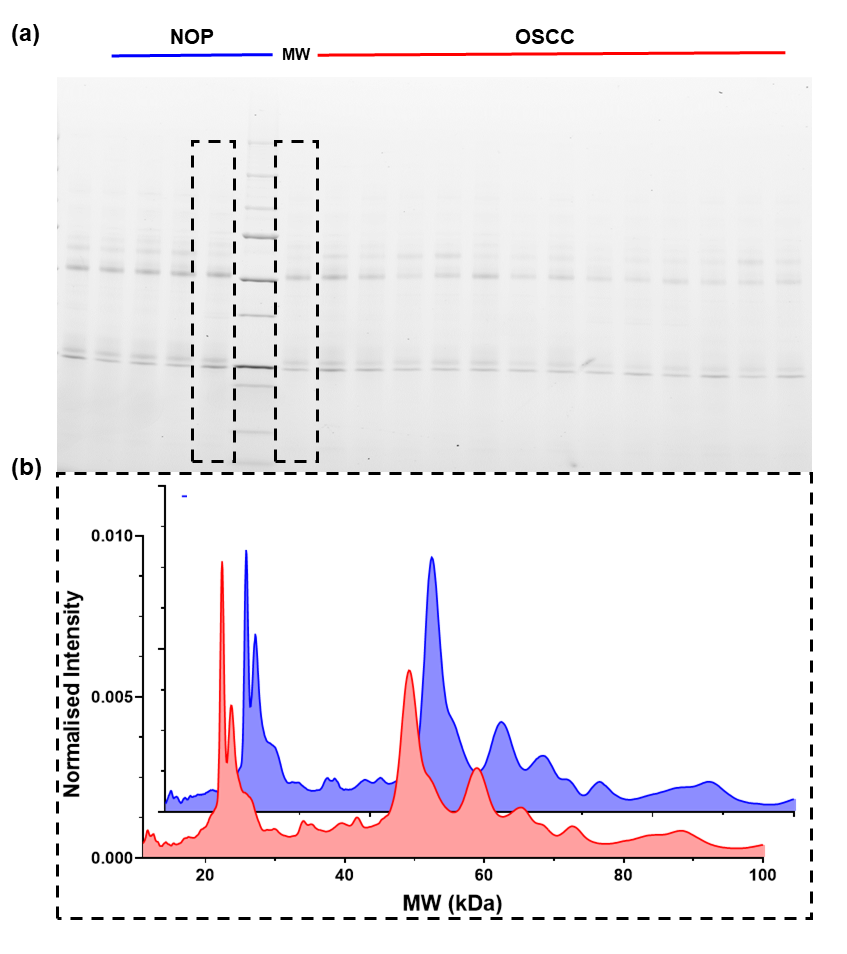

Supplement: Supplementary file 2 — Figure S2. After exposing graphene oxide (GO) sheets to human plasma (HP), a protein corona (PC) is generated. Following isolation of the PC through centrifugation and subsequent analysis using 1D SDS‐PAGE, an image of the gel is produced. In the gel image, each lane corresponds to the protein profile derived from an individual human subject, either a notoncological patient (NOP) or a patient affected by oral squamous cell carcinoma (OSCC). Representative 1‐dimensional (1D) profiles were derived by performing densitometric analysis on the two gel lanes highlighted by dashed boxes in the gel image. These profiles are presented for NOP (blue profile) and OSCC (red profile) within the molecular weight (MW) range of 10 to 100 kDa. [file ODI-31-3039-s002.png]
